# Leaching Behavior of As and Pb in Lead–Zinc Mining Waste Rock under Mine Drainage and Rainwater

**DOI:** 10.3390/toxics11110943

**Published:** 2023-11-20

**Authors:** Ziwen Guo, Jiejie Yang, Kewei Li, Jiaxin Shi, Yulong Peng, Emmanuel Konadu Sarkodie, Bo Miao, Hongwei Liu, Xueduan Liu, Luhua Jiang

**Affiliations:** Key Laboratory of Biometallurgy, School of Minerals Processing and Bioengineering, Ministry of Education, Central South University, Changsha 410083, China; kidgzw@hotmail.com (Z.G.); jiejieyang@csu.edu.cn (J.Y.); 15116475510@163.com (K.L.); 215611008@csu.edu.cn (J.S.); 225611009@csu.edu.cn (Y.P.); emmasark@csu.edu.cn (E.K.S.); miaobo@csu.edu.cn (B.M.); hongweiliu@csu.edu.cn (H.L.); xueduanliu@csu.edu.cn (X.L.)

**Keywords:** waste rock, lead–zinc mine, toxic metal(loid)s, occurrence characteristics, leaching behavior

## Abstract

At present, the pollution of arsenic (As) and lead (Pb) is becoming increasingly serious. The pollution caused by the release of As and Pb from lead–zinc mines has seriously affected the water and soil environment and threatened human health. It is necessary to reveal the release characteristics of As and Pb. The actual scene of mine drainage (MD) and rainwater (RW) leaching waste rocks is the one of the main reasons for the release of As and Pb. However, the leaching behavior of As and Pb in these waste rocks under MD and RW suffered from a lack of in-depth research. In this study, we investigated the occurrence of As and Pb in waste rocks (S1–S6) by using X-ray diffraction (XRD) and time-of-flight secondary ion mass spectrometry (TOF-SIMS), and then, the changes in As and Pb concentration and the hydrochemical parameter in leaching solution were systematically studied. Furthermore, the correlation between the release of As and Pb and mineral composition was also evaluated. Results showed that these waste rocks were mainly composed of carbonate and sulfide minerals. As and Pb were mainly bounded or associated with sulfide minerals such as arsenopyrite, pyrite, chalcopyrite, and galena in these waste rocks, and small parts of As and Pb were absorbed or encased by clay minerals such as kaolinite and chlorite. Under MD and RW leaching, the pH, redox potential (Eh), and electric conductivity (EC) of each waste rock tended to be consistent due to their buffering ability; the leachate pH of waste rocks with more carbonate minerals was higher than that of sulfide minerals. Both As and Pb were released most under MD leaching in comparison to RW, reaching 6.57 and 60.32 mg/kg, respectively, due to MD’s low pH and high Eh value. However, As in waste rock released more under alkaline conditions because part of the arsenic was in the form of arsenate. As and Pb release were mainly positively correlated with the proportions of sulfide minerals in these waste rocks. MD leaching significantly promoted the release of As and Pb from waste rocks, which would cause a great threat to the surrounding environment, and control measures were imperative. This paper not only reveals the As and Pb pollution mechanism around the lead–zinc mining area but also provides a theoretical basis for the prevention and control of As and Pb pollution in the future.

## 1. Introduction

Non-ferrous metal minerals resources are indispensable and play an important role in economic development [1]. In the process of the exploitation of mineral resources, a large amount of mining waste rock has been produced [2]. According to the report on National Mineral Resources *Conservation and Comprehensive Utilization (2020)* issued by the Ministry of Natural Resources of the People’s Republic of China, the stripping ratio of non-ferrous metal minerals ranged from 2.14 to 10.86 t/t in 2018 [3]; this means that a large number of mining wastes including tailings and waste rocks will be generated, although part of them can be used as resources. In the absence of strict environmental control, the weathered oxidation of these wastes will produce acid mine drainage (MD). Acid MD will lead to toxic metal(loid) pollution, posing a threat to the surrounding environment and human health [4].

Lead–zinc minerals are important non-ferrous metal mineral resources, being mostly distributed in parts of southern China such as the Yunnan, Hunan, and Fujian provinces [5]. The toxic metal(loid) pollution of lead–zinc mine areas usually contains Pb, As, Cd, Cr, Cu, Zn, and Ni [6,7,8]. Among these toxic metal(loid) elements, the most serious pollutants are As and Pb [9,10]. For example, Chenzhou is a typical lead–zinc mining area in Hunan Province, and the total concentrations of As and Pb in the soil of an abandoned lead–zinc mine in this area could reached up to 84.85 and 802.58 mg/kg, respectively, which were far beyond the national risk control values for development land soil [11]. Inner Mongolia is an important non-ferrous metal-producing area in northern China; both As and Pb in the soil around a typical lead–zinc mine in Chifeng, Inner Mongolia, China are seriously polluted, and the concentrations of As and Pb were found to be 82 and 600 mg/kg, respectively [12,13]. In Nigeria, Pb pollution in agricultural soil around the Ishiagu lead–zinc mining mine was serious, and the total concentration of Pb could reach up to 5305.5 mg/kg [14]. The pollution of As and Pb in the soil surrounding the lead–zinc mining area in northern Turkey was also serious and their concentrations surprisingly reached 1171 and 3725 mg/kg, respectively [15]. Therefore, it is urgent to study the release characteristics of As and Pb in the mining wastes at lead–zinc mines, which is of great significance for its environmental impact assessment and pollution control.

Usually, mining wastes are common toxic metal(loid)s bearing matrices that occur at lead–zinc mining sites [16]. The leaching behaviors of toxic metal(loid)s in different kinds of mining wastes are different due to the differences in the processing process [17]. For example, finer tailings have a larger contact area than the coarser characteristics of waste rocks, which makes it easier for them to oxidize and release toxic metal(loid)s [18,19]. Due to the tailings having a greater tendency to release toxic metal(loid)s than waste rocks, many studies have investigated their environmental risk, mobility, and safe disposal at present [20,21,22]. However, they found that tailings in most mining sites were often deposited in ponds to prevent seepage, and waste rocks were usually neglected and stored in open sites [3,23]. The pollution of As and Pb in waste rocks could also not be ignored. For example, the pollution of As and Pb in the soil of a lead–zinc mine in northern Guangxi, China mainly came from the mining waste rocks [24]. The waste rocks from underground polymetallic mines in Balya, Turkey caused a high concentration of As and Pb pollution in the Kocacay River during the wet season [25]. Due to the different net acid generation levels, redox reactions, and interfacial activity of toxic metal(loid)s in waste rocks, the environmental hazards caused by toxic metal(loid)s were also different. At present, only a few studies have investigated the release of toxic metal(loid)s from waste rocks. Therefore, the release behavior of toxic metal(loid)s in waste rocks at lead–zinc mines is worth exploring.

In past studies, laboratory static leaching tests and dynamic tests were commonly used to evaluate the release characteristics of toxic metal(loid)s in mining wastes [26,27,28]. Leaching solution pH, waste particle size, and solid–liquid ratio in the reaction system were the most studied factors influencing the release behavior of toxic metal(loid)s in mining wastes [29,30,31]. MD and rainwater (RW) were the most common media solutions that waste rocks were exposed to in mining areas. To reveal the oxidative dissolution behavior of waste rocks under MD and RW leaching, most studies have simulated RW and MD by using deionized water or acid solutions (nitric acid, acetic acid, sulfuric acid, hydrochloric acid) to leach waste rocks. The results have shown that all of them promoted the release of toxic metal(loid)s [32,33,34]. Using synthetic RW and acid MD as a leaching solution could partly reflect the oxidative dissolution of wastes in mining sites. However, the hydrochemistry of actual MD and RW is rather different from that of a simulation, leading to influencing the release of As and Pb in waste rocks. It is necessary to select MD and RW that exist in an actual scenario to explore the leaching behavior of As and Pb. Thus, a representative lead–zinc mine in Shanggao County, Jiangxi Province, China was investigated in this study and six waste rock samples were collected from this mine. The objective of this study was to analyze the release characteristics of As and Pb from waste rocks under MD and RW leaching using an inductively coupled plasma optical emission spectrometer (ICP-OES), time-of-flight secondary ion mass spectrometry (TOF-SIMS), and X-ray diffraction (XRD) and investigate the correlation between the release of As and Pb and the mineral components in the waste rocks through redundancy (RDA) analysis.

## 2. Materials and Methods

### 2.1. Chemicals

NaOH used in preparing the alkaline leaching solution was analytically pure and purchased from Sinopharm Chemical Reagent Co., Ltd, Shanghai, China. pH standard buffers (4.00, 6.86, and 9.18) used for pH meter calibration were obtained from Shanghai Lei Ci Co., Ltd, Shanghai, China. Electric conductivity (EC) standard solution (1413 μS/cm) used for EC meter calibration was provided by Mettler Toledo, Co., Ltd, Shanghai, China. The water for solution preparation was high-grade purified water (18.25 Ω/cm) prepared by Elga Purelab Chorus2, Lane End, High Wycombe, the UK.

### 2.2. Waste Rock Sample Collection and Pretreatment

**Figure 1:**
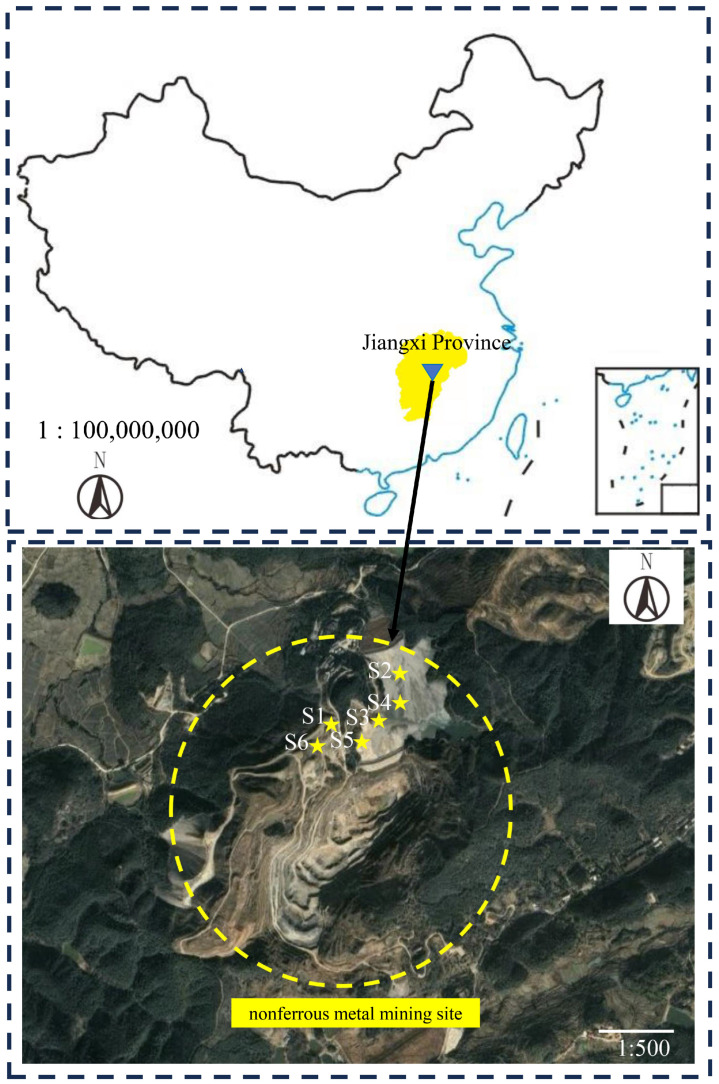
Location of the mining site and sampling overview. S1, S2, S3, S4, S5, and S6 were the six waste rock samples.

The waste rocks used in this study were obtained from a lead–zinc mining area in Shanggao County, Jiangxi Province, China (114°52′30″–114°55′00″ E, 28°09′00″–28°10′50″ N). According to the stacking time and waste rock type, six kinds of waste rock named S1–S6 were obtained from this mine. The overview sampling information is shown in Figure 1. After transporting samples to the laboratory, all waste rock samples were crushed and ground and then passed through 10-mesh (2.000 mm), 100-mesh (0.147 mm), and 200-mesh (0.074 mm) sieves. Then, these samples were sealed in zip-locked bags and stored in 4 °C refrigerator for later characterizations and leaching experiments.

### 2.3. Characterizations

The morphologies and element distributions of 10–mesh samples were analyzed by using scanning electron microscope (SEM, JEOL, JSK-6490LV, Tokyo, Japan) and X-ray spectrometer (EDS, EDAX, NEPTUNE TEXS HP, CA, USA) at a voltage of 20 kV [35]. Moreover, the element distributions of these samples were further exanimated using TOF-SIMS (ULVAC-PHI Inc., PHI nano TOF II, Kanagawa, Japan) at a voltage of 30 kV and an ion current of 2 nA [36]. Samples of the 200-mesh type were used for mineral composition measurement by using XRD (Bruker, D8 ADVANCE, Karlsruhe, Germany) with CuKa radiation (λ = 1.5406 nm) at a voltage of 40 kV and a current of 50 mA [37] The chemical compositions of samples were measured by using X-ray fluorescence spectrometer (XRF, Bruker, S4 Pioneer, Karlsruhe, Germany) with an accuracy of 0.02% and a limit of detection (LOD) of 0.5 mg/kg [38]. Samples of the 100-mesh type were digested by lead nitrate and acetic acid buffer solution (pH = 5) and then we detected the sulfate–sulfur through ethylene diamine tetra acetic acid titration [39]. The total carbon and sulfur determination of samples were investigated by using carbon sulfur analyzer (LECO, CS600, San Jose, CA, USA); the relative standard deviation (RSD), limit of blank (LOB), and limit of quantitation (LOQ) of total carbon and total sulfur were 0.5%, 0.3 mg/kg, and 0.6 mg/kg, respectively for both [40,41]. The content of sulfide–sulfur in samples was the difference between total sulfur and sulfate–sulfur [42].

### 2.4. Batch Leaching Tests

The leaching solutions including MD and RW were also collected from the lead–zinc mining area. In order to set up a comparison experiment, an alkaline leaching solution (AS) was prepared by adjusting the pH value of deionized water with NaOH. The solution chemical parameters of MD, RW, and AS are shown in Table 1. The leaching experiment was set as follows: 50 g of waste rocks (10-mesh) was added into MD, RW, and AS in conical flasks, and the liquid–solid ratio (mL/g) was set to 10:1. Then, the conical flasks were shaken at 25 °C and 180 r/min for 15 d in a vertical temperature oscillation incubator (Tianjin Lai Bo Terry Instrument Equipment Co., ZQPL-200, Tianjin, China). Samples were taken at 0.5, 1, 2, 3, 4, 6, 9, and 15 d, respectively. After static sedimentation, the leachates were provided to examinate the hydrochemistry parameters including the values of pH, redox potential (Eh), and EC and the total concentrations of As and Pb. The pH value was measured by using a digital pH meter (Leici, PHS-3E, Shanghai, China) according to standard procedure NY/T 1121.2−2006 [43]; the accuracy of this pH meter was 0.01 [43]. The Eh value was determined by using the digital pH meter (Leici, E-301-F, Shanghai, China) and Ag/AgCl electrodes with Pt electrodes according to standard procedure HJ 746−2015 [44]; the accuracy values of this pH meter and Ag/AgCl electrodes with Pt electrodes were 0.01 [44]. The EC value was exanimated by using conductivity meter (Mettler Toledo, FiveEasy Plus FE38, Zurich, Switzerland) based on HJ 802−2016 [45]; the accuracy of this conductivity meter was 0.1 [45]. The measurement of the total concentration of As and Pb was conducted with an ICP-OES (PerkinElmer, Avio500, Waltham, MA, USA); the ICP-OES had an accuracy of 0.001 mg/L. The RSD, LOB, and LOQ of As detected by ICP-OES were 0.5%, 0.053 mg/L, and 0.056 mg/L, respectively; these values for Pb, determined by ICP-OES, were 0.5%, 0.090 mg/L, and 0.091 mg/L, respectively [46].

### 2.5. Statistical Analysis

Statistical analyses were performed using Excel 2021 in Microsoft 365 (Office). All graphs were plotted by using Origin 2021b [43]. RDA analysis was performed and plotted by base R package vegan (v. 2.5.7) from R (v. 3.6.3). The release amount of As and Pb was calculated as follows: Cm = (C × V)/m. Here, Cm is the release amount (mg/kg), C is the concentration of As and Pb in leaching solutions (mg/L), V is the leaching solution volume (L), and m is the mass of waste rocks used for leaching (kg). To ensure the accuracy and precision of the determination, quality assurance (QA) protocol was followed via the utilization of a blank test and repeating the experiment three times. To ensure the quality control (QC), accuracy of ICP-OES determination of As and Pb concentrations was controlled by using standard solutions of As and Pb (0.1, 0.2, 0.5, 1, 5, 10, 20, and 100 mg/L) to recommend standard curve (R^2^ ≥ 0.999). We used 100 mg/L standard solutions of As (GSB 04−1714−2004) and Pb (GSB 04−1742-2004) [47]. The results showed that the concentrations of standard As and Pb solutions were 100.21 and 99.94 mg/L, respectively. To check the pH of the solutions, the 4.00 buffer was determined using a corrected pH meter, which gave a result of 3.99. To check the EC of the solutions, an EC buffer of 1413 μS/cm was determined using a calibrated EC meter, which gave a result of 1413 μS/cm. All data were recorded three times.

## 3. Results and Discussion

### 3.1. Mineral Component of Waste Rocks

**Figure 2:**
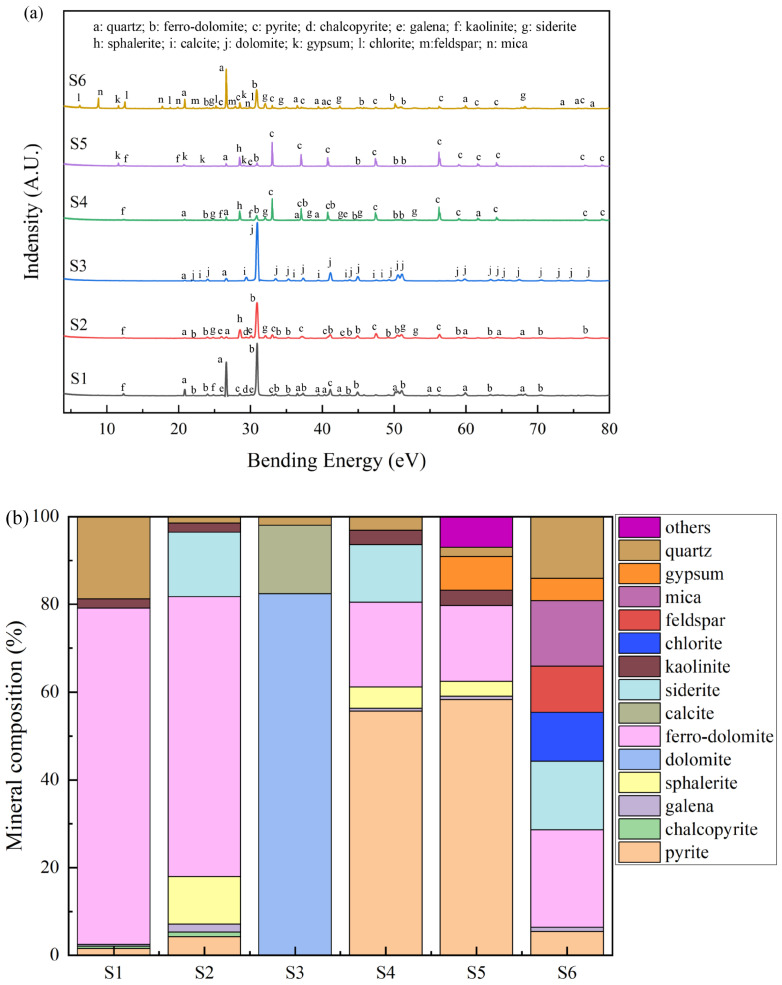
XRD spectra of waste rocks (**a**) and their mineral proportion (**b**).

The XRD patterns of the waste rocks and the proportions of identified minerals are shown in Figure 2. As shown, the mineral component of waste rocks could be classified into five categories: (i) sulfide minerals, including pyrite (FeS_2_), chalcopyrite (CuFeS_2_), sphalerite (ZnS), and galena (PbS); (ii) sulfate minerals such as gypsum (CaSO_4_); (iii) carbonate minerals, including dolomite [CaMg(CO_3_)_2_], ferro-dolomite [Ca(Mg,Fe)(CO_3_)_2_], siderite (FeCO_3_), and calcite (CaCO_3_); (iv) secondary clay minerals, including kaolinite [Al_4_(Si_4_O_10_)(OH)_8_] and chlorite [Y_3_(Z_4_O_10_](OH)_2_·Y_3_(OH)_6_. Y: Mg^2+^, Fe^2+^, Al^3+^, Fe^3+^. Z: Si^4+^ or Al^3+^]; and (v) primary aluminosilicates and silicate minerals, including quartz (SiO_2_), feldspar (KAlSi_3_O_8_, NaAlSi_3_O_8_, and CaAl_2_Si_2_O_8_), and mica [KAl_2_(AlSi_3_O_10_)(OH)_2_].

As and Pb are often associated with sulfide minerals and are easily released in acid MD by the oxidation and weathering of waste rocks [48]. The acid production abilities of sulfide minerals were different. Arsenopyrite, pyrite, and chalcopyrite (leachate pH = 1.68, 1.82, and 2.73, respectively) had higher acid production ability, whereas sphalerite and galena (leachate pH = 4.32, and 4.07, respectively) had lower production ability [49]. As shown in Figure 2b, S4 and S5 were mainly composed of sulfide minerals. Their proportion reached 61.2 and 62.5%, respectively. However, the proportions of sulfide minerals in S1, S2, S3, S6 were only 2.5, 18.0, 0, and 11.5%, respectively. This indicated that S4 and S5 had higher acid production ability than other waste rocks, and As and Pb were more easily released in these waste rocks through oxidation and dissolution.

Carbonate mineral and siliceous minerals in the waste rocks can neutralize acid produced by the sulfide mineral oxidation [50]. It has been reported that the waste rocks without carbonate minerals have a greater potential to produce high concentrations of metal(loid)s than those containing carbonate minerals after acid MD leaching [51]. As shown in Figure 2b, the proportions of carbonate minerals in S1, S2, S3, S4, S5, and S6 were 76.6, 78.4, 98.0, 32.4, 17.2, and 37.9%, respectively. S1, S2, and S3 contained more carbonate minerals than S4, S5, and S6. This suggested that S1, S2, and S3 had stronger acid neutralization ability, potentially resulting in reducing the production of MD and the release of As and Pb. Clay minerals have the ability to absorb or encapsulate metal(loid) ions including As and Pb [52]. It has been reported that clay could absorb As via ligand exchange and electrostatic adsorption and absorb Pb through competitive adsorption onto non-specific sites on the surface of clay [35,53,54]. Figure 2b shows that the contents of clay minerals in all waste rocks were very low (2.1, 2.1, 0, 3.3, and 3.5%) except in S6 (11.1%). This meant that As and Pb released in S6 would be re-adsorbed to waste rock through clay mineral adsorption, leading to a relatively low release amount of S6 [55].

### 3.2. Chemical Composition of Waste Rocks

The chemical composition of these waste rocks is shown in Table 2. As seen, the proportions of alkaline oxide including CaO and MgO in S1, S2, and S3 were less than that in S4, S5, and S6. This suggested that the acid neutralization ability of S1, S2, and S3 might be lower than that of S4, S5, and S6 due to the hydrogen protons caching the properties of the alkaline oxides [56]. However, the acid neutralization ability of S1, S2, and S3 might have been improved due to the higher carbonate mineral contents as shown in the XRD results (Figure 2). In addition, it had been reported that Fe_2_O_3_, Al_2_O_3_, and MnO have adsorption ability to adsorb metal(loid)s including Pb and As [57,58,59]. The amounts of Fe_2_O_3_ and Al_2_O_3_ in S1, S2, and S3 were higher than that in S4, S5, and S6. S1 and S2 contained more Fe_2_O_3_, reaching 73.50 and 77.43%, respectively; the content of Al_2_O_3_ in S3 was 22.96%, which was higher than that in the other waste rocks. This indicated that Ca and Mg of the ferro-dolomite in S1, S2, and S3 were replaced by Fe and Al [60]. As a result, S1, S2, and S3 had low CaO and MgO content but high Fe_2_O_3_ and Al_2_O_3_ content. This suggested that S1, S2, and S3 might have greater adsorption potential for As and Pb.

The total carbon content values of S1, S2, and S3 (6.75–10.80%) were higher than those of S4, S5, and S6 (0.71–3.56%). This indicated that S1, S2, and S3 contained more carbonate minerals, which was consistent with the XRD results as shown in Figure 2. The total sulfur contents of S2, S4, and S5 were 42.54%, 48.60%, and 49.92%, respectively, which were much higher than those of S1, S3, and S6, which reached 1.42, 0.17, and 3.78%, respectively. Most of them were in the form of sulfide–sulfur, except in S3. This sulfide–sulfur might comprise sulfide minerals such as pyrite, chalcopyrite, and galena, according to the results shown in Figure 2. This suggested that the waste rocks of S2, S4, and S5 contained large amounts of sulfide–sulfur and had a stronger ability to produce acid, leading to the release of more As and Pb from these waste rocks [61]. The proportions of As and Pb in these waste rocks were 0.06% and 1.99%, respectively. These values exceeded the national risk control value [62], potentially posing a significant threat to the surrounding environment.

### 3.3. Morphology and Element Distribution of Waste Rocks

**Figure 3:**
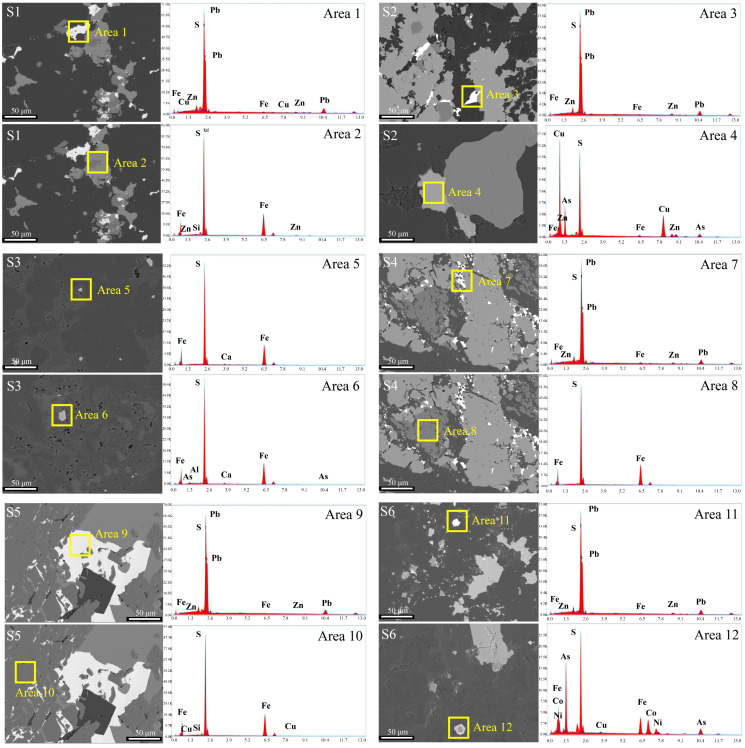
SEM-EDS images of waste rocks. Area 1–12 were the mineral components where As and Pb may exist on six waste rock samples (S1–S6) determined by SEM-EDS.

The morphology and element content of the waste rocks are illustrated in Figure 3. All the waste rocks were heterogeneous and contained various mineral components. All of them were quartz and dolomite [63]. S4, S5, and S6 had more irregularly shaped particles than S1, S2, and S3. This indicated that S4, S5, and S6 had larger surface areas in contact with the leaching solution and it was easier for them to oxidize and release As and Pb. EDS results are shown in Figure 3; Pb exists in all waste rocks except S3. The results were similar to those of the XRF (Table 2). As reflected by the EDS results, Pb concentrations in S1, S2, S4, S5 and S6 were 85.80, 84.14, 84.27, 87.64, and 89.36, respectively. Meanwhile, the S concentration were 11.08, 12.20, 11.42, 12.06, and 9.52%, respectively. Pb was associated with S, which indicated that Pb in these waste rocks might have been in the form of galena. In addition, As was only found in S2, S3, and S6, as exhibited by EDS, in concentrations of 19.68%, 1.31% and 31.23%, respectively. EDS results showed that As coexisted with S, Fe, Cu, Al, Ca, and Si. This suggested that As in these waste rocks might be associated with pyrite or chalcopyrite, which was consistent with the XRD results. As(III) and As(-I) would substitute Fe(II) and S_2_(–II), respectively in pyrite and chalcopyrite [64]. A small part of As in these waste rocks was adsorbed or coated by clay minerals like kaolinite and chlorite in the form of arsenate [54].

**Figure 4:**
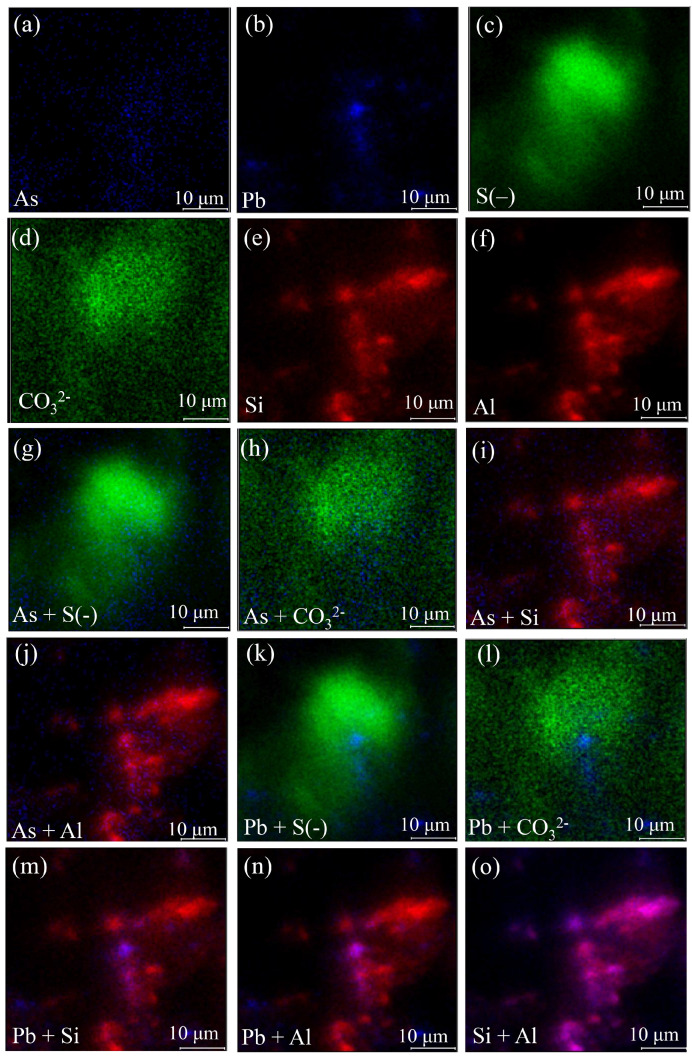
TOF-SIMS images in S2: (**a**–**f**) distribution of As, Pb, S(–), CO_3_^2−^, Si, and Al; (**g**–**j**) overlap of As with S(–), CO_3_^2−^, Si, and Al, respectively; (**k**–**n**) overlap of Pb with S(–), CO_3_^2−^, Si, and Al, respectively; (**o**) overlap of Si with Al. The color is simply to distinguish the distribution of different elements.

An S2 sample was chosen for TOF-SIMS analysis to further study the distribution characteristics of As and Pb. Figure 4 shows selected secondary ion images acquired for the S2 sample. As shown in Figure 4a–f, As, Pb, S(–), CO_3_^2−^, Si, and Al in S2 were found. CO_3_^2−^ were uniformly distributed in S2, while As, Pb S(–), Si, and Al were enriched. As and Pb had low signal intensity, while S(–), CO_3_^2−^, Si, and Al had high signal intensity. This was consistent with the results of the XRF shown in Table 2. The overlap of As and S(–), CO_3_^2−^, Si, and Al is illustrated in Figure 4g–j. The signal of As exhibited a high overlap with S, indicating that As might be associated with sulfide minerals such as pyrite, chalcopyrite, and arsenopyrite [65]. These sulfide minerals conformed with the results of SEM-EDS. The signal of As showed some overlap with Al and Si, and Al highly overlapped with Si, as shown in Figure 4o. This indicated that some parts of As in S2 might have been adsorbed or encased in clay minerals [66,67]. There was little overlap between As and CO_3_^2-^. This suggested that there was a low correlation between As and carbonate minerals in these waste rocks. Figure 4k–n show the overlap of Pb and S(–), CO_3_^2−^, Si, and Al. The overlap of Pb’s signal with S(–), CO_3_^2−^, Si, and Al was similar to that of As. The signal of Pb exhibited a high overlap with S(–). This suggested that Pb in S2 was mainly composed of galena [68]. There was some overlap between Pb, Al, and Si. This indicated that a part of Pb was adsorbed or encased with clay minerals [69]. Pb and CO_3_^2−^ had little overlap. This indicated a very low correlation between Pb and carbonate minerals.

### 3.4. The Changes in pH, Eh, and EC Values of Leaching Solutions

**Figure 5:**
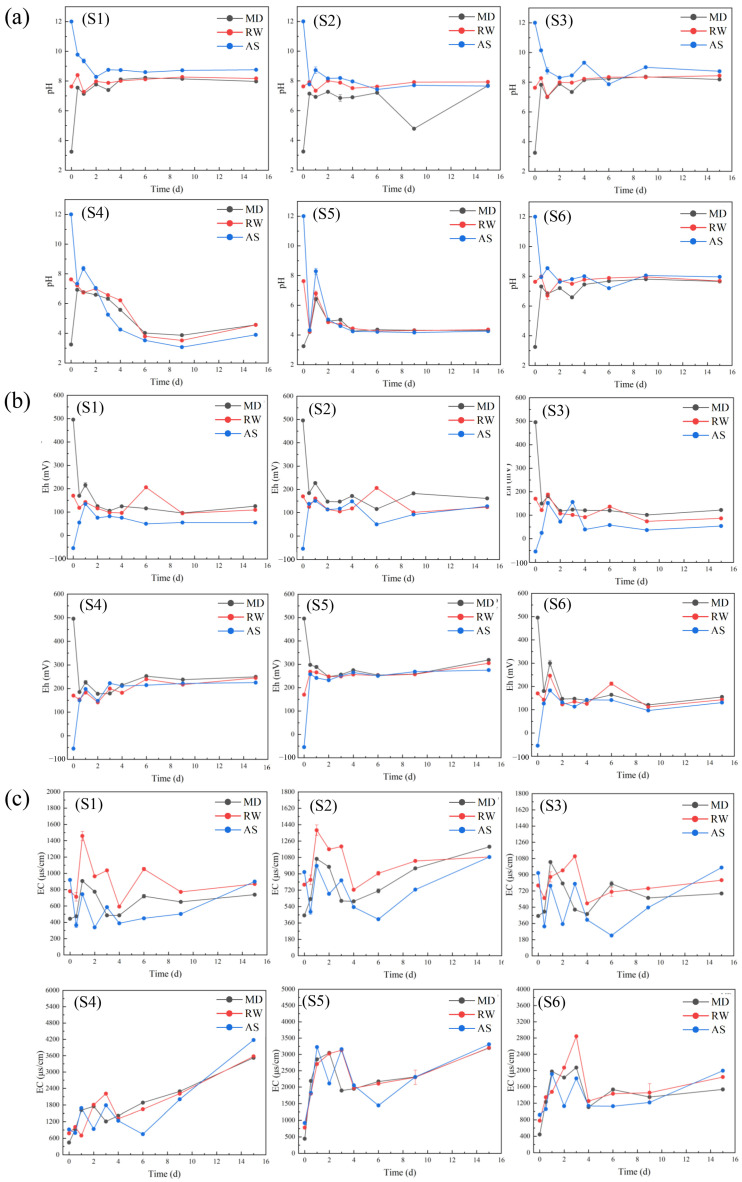
The pH (**a**), Eh (**b**), and EC (**c**) values in leaching solution. S1–S6 are the samples of waste rock.

The values of pH, Eh, and EC of the leaching solutions during waste rock leaching by MD, RW, and AS are illustrated in Figure 5. Their values remained stable after leaching by MD, RW, and AS. This might be attributed to the acid–base buffer characteristics of these waste rocks due to the existence of sulfide minerals and carbonate minerals [49,50]. Carbonate mineral dissolution could neutralize the acid produced by sulfide mineral dissolution [70]. As shown in Figure 5a, the pH values of leaching solutions in S1–S3 samples ranged from 7.5 to 8.5 when leached by MD, RW, and AS. This could be attributed to the high acid neutralizing ability of the dolomite, ferro-dolomite, siderite, calcite, CaO, and MgO and the weak acid production ability of the sulfide minerals. Therefore, although a small amount of sulfide minerals was oxidized to produce acid in S1–S3, acid would be neutralized quickly by these carbonate minerals and alkaline oxides. The pH values of S4–S5 in MD, RW, and AS decreased to 3.90–4.57 after 15 d of leaching. The net acid production ability of S4 and S5 was high, due to their having greater amounts of sulfide minerals (reaching 61.2 and 62.5%, respectively). The pH value of the final leachates in S6 was maintained in the range of 7.0–8.0. The content of carbonate minerals was slightly greater than that of sulfide minerals. This indicated that the acid neutralization ability was slightly greater than the acid production ability.

The Eh value of the leaching solution could reflect the redox state in the leaching process [71]. The initial Eh value of MD (495.67 mV) was much higher than those of RW (170.00 mV) and AS (−54.67 mV). This meant that the oxidation potential of MD was much higher than those of RW and AS, resulting in the acceleration of the oxidative dissolution of sulfide minerals in waste rocks when leached by MD. As shown in Figure 5b, Eh decreased in MD leaching in all waste rocks at 1 d; Eh was unchanged in RW leaching at 1 d, while it increased in AS leaching. The Eh values of the three leaching solutions became increasingly similar as leaching time increased. This might be ascribed to the waste rocks having the same redox conditions due to the convergence of pH values during leaching [72]. The Eh of the MD leaching solution was higher than those of RW and AS, indicating that As and Pb were more easily released during MD leaching. Moreover, the Eh values of S1, S2, S3, S4, S5, and S6 in MD decreased from 495.67 to 125.33, 161.33, 122.00, 249.33, 318.67, and 155.00 mV, respectively, after 15 d leaching; the Eh values in RW after 15 d were 109.33, 124.00, 86.67, 245.00, 304.67, and 143.00 mV, respectively. The Eh values of these waste rocks increased from −54.67 to 55.00, 127.33, 54.00, 225.33, 275.33, and 130.67 mV, respectively in AS leaching for 15 d. The Eh values of S4 and S5 were higher than those of the other waste rocks. This suggested that the sulfide minerals of S4 and S5 could be easily oxidized, releasing more As and Pb. The Eh values of these waste rocks in MD leaching were higher than those in RW leaching and AS leaching [73]. This indicated that waste rocks were more easily oxidized in MD leaching than in RW and AS.

EC can indirectly reflect the concentration of metal(loid) ions in leaching solutions [74]. The initial EC values of MD, RW, and AS were 443.23, 780.90, and 919.63 μS/cm, respectively. As shown in Figure 5c, the EC values of the leaching solutions in each waste rock tended to be consistent in the middle and late stages of leaching and showed a rising trend. This could be attributed to the release of the As and Pb in waste rocks [74]. The EC values of S1, S2, S3, S4, S5, and S6 after 15 d reached 739.23, 1197.33, 691.97, 3521.00, 3197.00, and 1534.67 μS/cm, respectively in MD leaching; 870.60, 1084.67, 840.00, 3574.00, 3205.67, and 1837.00 μS/cm, respectively in RW leaching; and 899.93, 1084.67, 979.93, 4176.33, 3309.00, and 1993.00 μS/cm, respectively in AS leaching. The EC values of S1, S2, S3, and S6 were much lower than S4 and S5. This indicated that the As and Pb release abilities of S2 and S6 might be higher than those of S1 and S3 and lower than those of S4 and S5 in these leaching solutions.

### 3.5. The Change in Total Concentrations of As and Pb in Leaching Solutions

**Figure 6:**
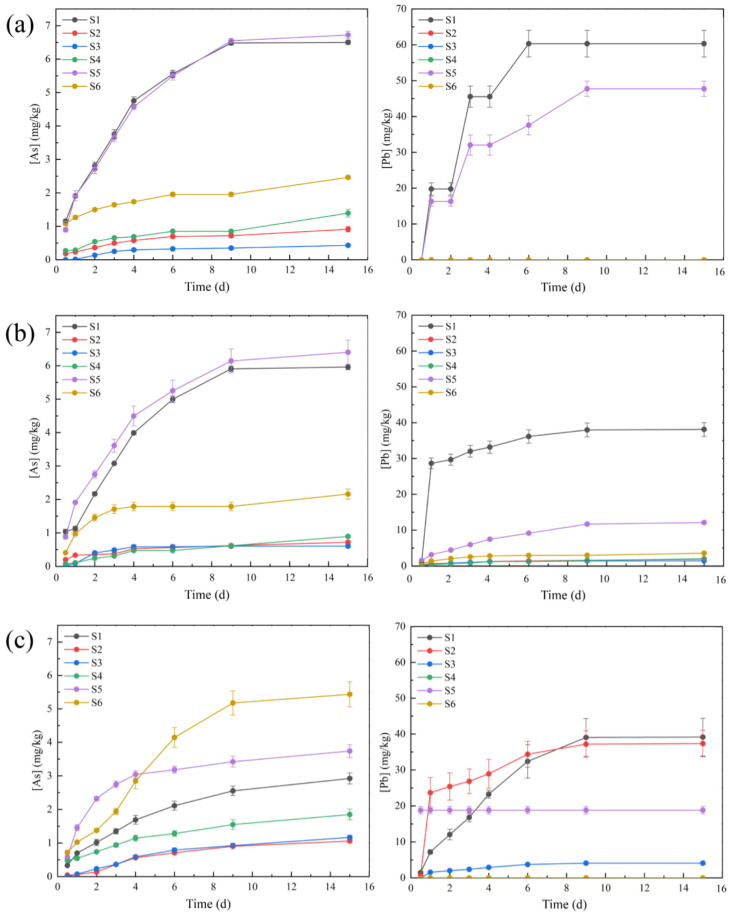
Releasing concentrations of As and Pb under MD (**a**), RW (**b**), and AS (**c**) leaching. On the left is the release of As in waste rock, and on the right is the release of Pb in waste rock.

Figure 6 shows the release of As and Pb in S1–S6 under MD, RW, and AS. Figure 6a shows the As and Pb released in S1–S6 under MD leaching. As and Pb in S1 and S5 were released the most in these waste rocks, reaching 6.50, 60.32, and 6.57, 47.72 mg/kg, respectively, as shown in Figure 5. The pH of S1 was maintained at about 8 and the Eh was maintained at about 100 mV due to the dissolution of ferro-dolomite, and the solution was in a low-oxidation environment. This indicated that As might have existed in the form of arsenate in S1 [75]. S1 has a high Pb release due to its high total Pb content (0.33%). S5 was mainly composed of pyrite and galena; the pH and Eh of the leaching solution were maintained at 4 and 200 mV, respectively in MD leaching. This meant that sulfide minerals were more easily dissolved and released As and Pb under acidic conditions [76]. The release amounts of As and Pb in S2, S3, S4, and S6 were very low, and values were observed at 0.91 and 0, 0.43 and 0, 1.39 and 0, and 2.46 and 0 mg/kg, respectively. This might have been because the total As and Pb contents in S2, S3, S4, and S6 were very low or the forms of As and Pb in these waste rocks were not easily released [77]. Figure 6b shows the release of As and Pb in S1–S6 under RW leaching. The leaching results of RW were similar to those of MD. S1 and S5 had the highest As release levels, reaching 5.96 and 6.40 mg/kg, respectively under RW leaching. The release of Pb under RW leaching was much lower than that under MD leaching; the concentrations of S1 and S5 under RW leaching were 38.11 and 12.19 mg/kg, respectively. This indicated that Pb was more likely to be released when the initial condition was acidic. The release of As and Pb in these waste rocks under AS leaching is shown in Figure 6c. The release levels of S2, S3, S4, and S6 was much higher than under MD and RW leaching, reaching 1.06, 1.16, 1.85, and 5.43 mg/kg. This indicated that the As in these waste rocks could mainly exist in the form of anions, which were easy to release under alkaline conditions. The release of Pb in AS leaching was also much higher than in RW leaching, but less than in MD leaching. This suggested that the release of Pb could increase under alkaline conditions [78]. In general, both As and Pb were more likely to be released under acidic and alkaline conditions, with the acidic conditions being significantly better.

### 3.6. Correlation between Release of As and Pb and Mineral Composition

**Figure 7:**
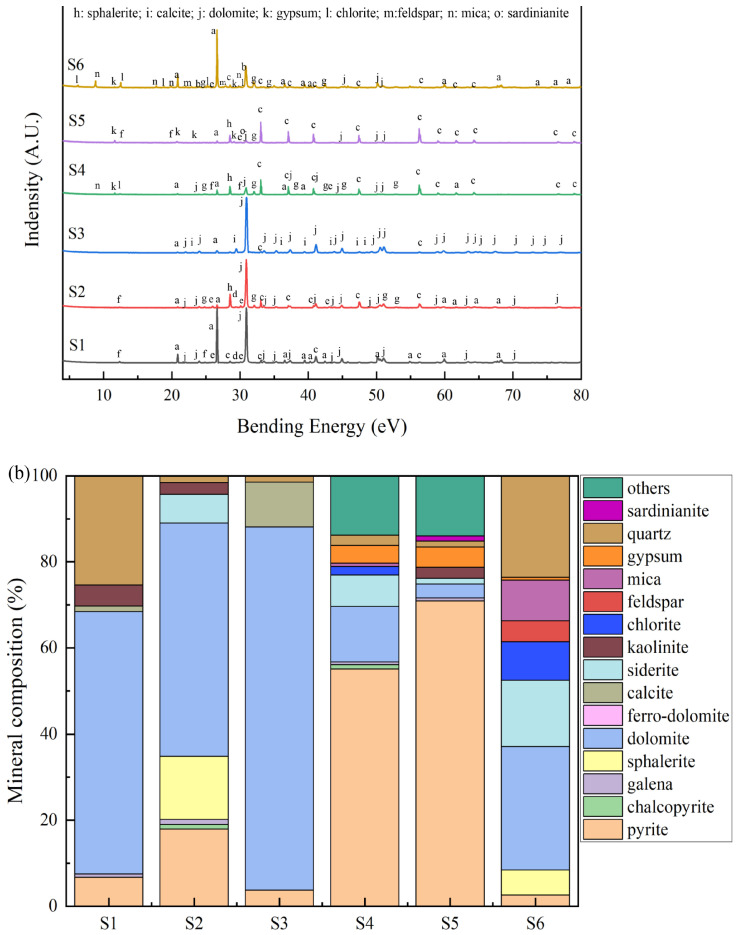
The XRD spectra of waste rocks after MD leaching (**a**) and their mineral proportions (**b**).

**Figure 8:**
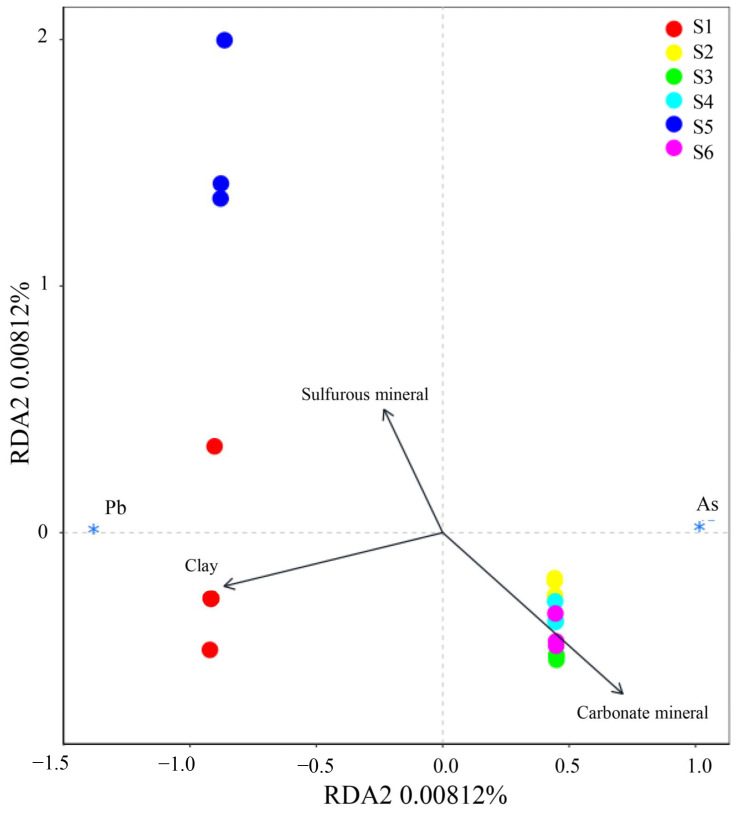
Ordination diagrams from RDA of releasing concentrations of As and Pb and the mineral composition in waste rocks during MD leaching. The * in the figure only indicates the position of As and Pb, and has no other meaning.

This was selected for analysis, due to the significant release of As and Pb obtained in MD leaching. Figure 7 shows the XRD patterns of waste rocks after MD leaching and the proportions of identified minerals. As shown in Figure 2 and Figure 7, the ferro-dolomite of S1, S2, S4, S5, and S6 was transformed into dolomite by deferrization under MD leaching. This indicated that the oxidation and dissolution of waste rocks would be accelerated due to the presence of Fe(III). Moreover, the content of gypsum in S4 increased from 0 to 4.1%, and anglesite (PbSO_4_) occurred in S5 after MD leaching. These suggested that sulfide minerals in waste rocks have been oxidized to generate secondary sulfate minerals. Furthermore, the carbonate mineral content in S1–S5 decreased from 76.6%, 78.4%, 98.0%, 32.4%, and 17.2% to 62.2%, 60.9%, 94.8%, 20.2%, and 4.6% whereas the content of sulfur minerals increased in all waste rocks. This indicated that carbonate minerals dissolve under MD leaching, leading to an increase in the proportions of sulfur minerals. The correlation between the release concentrations of As and Pb under MD leaching and the mineral composition of waste rocks was obtained via RDA analysis (Figure 8). The sulfide mineral composition in waste rocks was positively correlated with the release concentration of As and Pb in the leaching solution [79]. This indicated that the release of As and Pb mainly came from the sulfide minerals in these waste rocks. When the waste rock was soaked in acid MD, metal sulfide minerals such As pyrite were dissolved. Fe and As were also dissolved in the leaching solution. Fe(II) was oxidized to Fe(III), which accelerated the dissolution of other sulfide minerals such as galena [80]. It was found that the high pollution of As and Pb in a lead–zinc mining area occurred due to the oxidation dissolution of sulfide minerals in waste rocks [79,81]. Carbonate and clay minerals in waste rocks were negatively correlated with the release concentration of As and Pb. The negative correlations of carbonate minerals with Pb releasing were lower than with As releasing. The dissolution of carbonate minerals would promote the reaction of Fe(II) with the thioarsenite to form arsenopyrite, thereby reducing the release of As [82]. The negative correlation between the dissolution of clay minerals and the release of Pb was greater than that of As. Part of the As was adsorbed or encased in clay minerals in the form of anions [66,67]. This indicated that more Pb than As would be released during the dissolution of clay minerals, but the opposite would be true in the dissolution of clay minerals.

## 4. Conclusions

The findings showed that waste rocks in lead–zinc mine were mainly composed of carbonate minerals and sulfide minerals. The net acid production potential of S4 and S5 was greater due to their having a higher proportion of sulfide minerals and lower content of carbonate minerals. The main occurrence form of As was arsenopyrite, and a small part was associated with pyrite and chalcopyrite. However, the main occurrence form of Pb was galena. In addition, small amounts of As and Pb were adsorbed or encased by clay minerals. During the leaching of MD and RW, the pH, Eh and EC of each waste rock tended to be similar since each of these waste rocks had an acid–base neutralization ability. The leachate pH values of S1, S2, S3, and S6 were significantly higher than those of S4 and S5 due to their having a higher content of carbonate minerals and acid neutralization ability. Because MD had a higher acidity and Eh value, As and Pb were more easily released during MD leaching when compared to RW. Part of the As existed in the form of arsenate and was easier to release under alkaline conditions. Furthermore, the release of As and Pb in MD leaching showed a positive correlation with the change in sulfide minerals in these waste rocks but a negative correlation with clay minerals and carbonate minerals. This suggested that the release of As and Pb in waste rocks was affected by the environment and mineral composition and their physical and chemical properties. At present, As and Pb pollution control is still urgent. This paper has provided the basic data for subsequent pollution control and prevention. Future pollution control should focus on preventing the oxidation and dissolution of sulfide minerals in waste rocks.

## Figures and Tables

**Table 1:** Initial chemical parameters of leaching solutions. MD is ‘mine drainage’, RW is ‘rain water’, AS is ‘alkaline solution’, Eh is ‘redox potential’, and EC is ‘electric conductivity’.

Chemical Parameter	MD	RW	AS
pH	3.24	7.63	12.00
Eh (mV)	495.67	170.00	−54.67
EC (μS/cm)	443.23	780.90	919.63
As	N.D.	N.D.	N.D.
Pb	N.D.	N.D.	N.D.

N.D.: Not detected.

**Table 2:** Chemical composition of waste rocks provided. The oxides, As, and Pb were determined using XRF. Total carbon and total sulfur were determined using a carbon sulfur analyzer. Sulfide–sulfur was determined through titration. Sulfate–sulfur was calculated using the difference between total sulfur and sulfide–sulfur.

Chemical Composition	S1	S2	S3	S4	S5	S6
Na_2_O	0.11%	0.78%	1.31%	N.D.	1.51%	N.D.
MgO	2.45%	1.53%	2.88%	17.66%	23.97%	29.44%
Al_2_O_3_	4.85%	4.44%	22.96%	6.99%	2.96%	0.62%
SiO_2_	7.79%	5.70%	32.93%	30.24%	3.87%	3.70%
P_2_O_5_	0.07%	0.07%	0.26%	0.06%	0.09%	N.D.
K_2_O	0.06%	0.02%	2.84%	0.17%	0.06%	0.04%
CaO	4.24%	4.05%	7.36%	30.22%	41.41%	56.84%
MnO	0.43%	0.23%	0.79%	0.70%	1.69%	0.57%
Fe_2_O_3_	73.50%	77.42%	23.82%	8.82%	1.13%	4.83%
As	0.02%	0.03%	N.D.	0.03%	0.04%	0.06%
Pb	0.33%	1.99%	0.03%	0.51%	0.71%	0.34%
Total carbon	6.75%	8.66%	10.80%	1.55%	0.71%	3.56%
Total sulfur	1.42%	42.54%	0.17%	48.60%	49.92%	3.78%
Sulfide–sulfur	1.01%	41.23%	0.01%	47.40%	46.14%	2.29%
Sulfate–sulfur	0.41%	1.31%	0.16%	1.20%	3.78%	1.49%

N.D.: Not detected.

## Data Availability

The original data presented in the study are included in the article; further inquiries can be directed to the corresponding author.
